# Pharmacokinetic-Pharmacodynamic Characterization of Omadacycline against Haemophilus influenzae Using a One-Compartment *In Vitro* Infection Model

**DOI:** 10.1128/AAC.02265-19

**Published:** 2020-05-21

**Authors:** Brian D. VanScoy, Elizabeth A. Lakota, Haley Conde, Jennifer McCauley, Lawrence Friedrich, Judith N. Steenbergen, Paul G. Ambrose, Sujata M. Bhavnani

**Affiliations:** a Institute for Clinical Pharmacodynamics, Inc., Schenectady, New York, USA; b Paratek Pharmaceuticals, Inc., Boston, Massachusetts, USA

**Keywords:** omadacycline, *Haemophilus influenzae*, *in vitro* infection model, pharmacokinetics-pharmacodynamics

## Abstract

Omadacycline is a novel aminomethylcycline with activity against Gram-positive and -negative organisms, including Haemophilus influenzae, which is one of the leading causes of community-acquired bacterial pneumonia (CABP). The evaluation of antimicrobial agents against H. influenzae using standard murine infection models is challenging due to the low pathogenicity of this species in mice. Therefore, 24-h dose-ranging studies using a one-compartment *in vitro* infection model were undertaken with the goal of characterizing the magnitude of the ratio of the area under the concentration-time curve (AUC) to the MIC (AUC/MIC ratio) associated with efficacy for a panel of five H. influenzae isolates. These five isolates, for which MIC values were 1 or 2 mg/liter, were exposed to omadacycline total-drug epithelial lining fluid (ELF) concentration-time profiles based on those observed in healthy volunteers following intravenous omadacycline administration. Relationships between change in log_10_ CFU/ml from baseline at 24 h and the total-drug ELF AUC/MIC ratios for each isolate and for the isolates pooled were evaluated using Hill-type models and nonlinear least-squares regression. As evidenced by the high coefficients of determination (*r*^2^) of 0.88 to 0.98, total-drug ELF AUC/MIC ratio described the data well for each isolate and the isolates pooled. The median total-drug ELF AUC/MIC ratios associated with net bacterial stasis and 1- and 2-log_10_ CFU/ml reductions from baseline at 24 h were 6.91, 8.91, and 11.1, respectively. These data were useful to support the omadacycline dosing regimens selected for the treatment of patients with CABP, as well as susceptibility breakpoints for H. influenzae.

## Abstract (precis)

Omadacycline is a novel aminomethylcycline with activity against Gram-positive and -negative organisms, including Haemophilus influenzae, which is one of the leading causes of community-acquired bacterial pneumonia (CABP). The evaluation of antimicrobial agents against H. influenzae using standard murine infection models is challenging due to the low pathogenicity of this species in mice. Therefore, 24-h dose-ranging studies using a one-compartment *in vitro* infection model were undertaken with the goal of characterizing the magnitude of the ratio of the area under the concentration-time curve (AUC) to the MIC (AUC/MIC ratio) associated with efficacy for a panel of five H. influenzae isolates.

## INTRODUCTION

Community-acquired bacterial pneumonia (CABP) is a major cause of morbidity and mortality worldwide (1–3). After Streptococcus pneumoniae, Haemophilus influenzae is a leading cause of CABP (1, 4–6). Rates of ampicillin resistance among H. influenzae isolates collected worldwide have been reported to range from 6.38 to 43.0% (7), while 34.5% of isolates collected in the United States and Europe were reported to be trimethoprim-sulfamethoxazole nonsusceptible (8). Despite these data, susceptibility to other agents, including amoxicillin-clavulanic acid, ceftriaxone, levofloxacin, tigecycline, and tetracycline, has remained high (8). However, higher resistance rates have been reported in patients with cystic fibrosis, for whom antibiotic use is high and isolates have demonstrated resistance to macrolides (9, 10).

Omadacycline, a novel tetracycline known as an aminomethylcycline, was synthesized by the chemical modification of minocycline (11) and demonstrates *in vitro* activity against the most common Gram-positive and -negative pathogens associated with CABP, including H. influenzae (8). Intravenous (i.v.) and oral formulations of omadacycline were recently approved by the U.S. Food and Drug Administration for the treatment of patients with CABP (12). As part of the omadacycline drug development program, *in vivo* studies were carried out to characterize the pharmacokinetics-pharmacodynamics (PK-PD) of omadacycline against Staphylococcus aureus and S. pneumoniae (13, 14). However, PK-PD evaluations for H. influenzae using *in vivo* infection models are challenging. This is due primarily to the fact that H. influenzae does not grow well and auto-clears in murine infection models (15, 16), even the neutropenic thigh infection model, the workhorse infection model that has been used to characterize the PK-PD of essentially all classes of antimicrobial agents to date (17). In such cases, *in vitro* infection models have allowed for the characterization of the PK-PD of a given antimicrobial agent against H. influenzae (18–21).

Herein, we describe the results of a series of *in vitro* studies evaluating the activity of omadacycline against H. influenzae. The objectives of these studies were 2-fold. The first objective was to evaluate the frequency of mutation of clinical H. influenzae isolates to omadacycline. As the ratio of the area under the concentration-time curve from 0 to 24 h to the MIC (AUC/MIC ratio) is generally considered to be the PK-PD index most closely associated with efficacy for tetracyclines (22–25), the second objective was to determine the magnitude of the omadacycline AUC/MIC ratio associated with efficacy for a panel of H. influenzae isolates evaluated using a one-compartment *in vitro* infection model. Given the importance of considering effect site drug exposures (26–28), which for patients with CABP is epithelial lining fluid (ELF), the studies described herein were carried out in the context of omadacycline total-drug ELF concentration-time profiles to characterize AUC/MIC ratio targets associated with efficacy.

## RESULTS

### *In vitro* susceptibility studies.

The omadacycline MIC values for the panel of five H. influenzae isolates are presented in Table 1. Omadacycline MIC values ranged from 1 to 2 mg/liter and from 0.5 to 1 mg/liter for the microbroth and agar dilution methods, respectively.

**TABLE 1:** Omadacycline susceptibility results for the five H. influenzae isolates evaluated in the *in vitro* studies

H. influenzae isolate	Omadacycline MIC (mg/liter) from:	
	Microbroth dilution	Agar dilution
ATCC 49247	2	1
437	1	0.5
543	2	1
2696	2	1
10929	2	1

### Mutation frequency studies.

The inocula evaluated in the frequency of resistance studies failed to produce or identify a drug-resistant subpopulation for all isolates evaluated, as presented in Table 2. The lack of colonies found to grow on the antibiotic-supplemented agar plate suggests that the frequency of mutations resulting in omadacycline resistance was less than that of the inocula (2.9 × 10^9^ CFU/ml) examined.

**TABLE 2:** Average frequencies of omadacycline resistance for the five H. influenzae isolates at 48 h postinoculation based on data from two sets of studies

H. influenzae isolate	Baseline omadacycline agar MIC (mg/liter)	Inoculum (CFU/ml)	48-h observation	
			3× MIC	5× MIC
ATCC 49247	1	1.3 × 10^9^	<7.7 × 10^−10^	<7.7 × 10^−10^
437	0.5	2.3 × 10^9^	<4.3 × 10^−10^	<4.3 × 10^−10^
543	1	2.1 × 10^9^	<4.8 × 10^−10^	<4.8 × 10^−10^
2696	1	2.9 × 10^9^	<3.5 × 10^−10^	<3.5 × 10^−10^
10929	1	1.7 × 10^9^	<5.9 × 10^−10^	<5.9 × 10^−10^

### One-compartment *in vitro* infection model.

**FIG 1:**
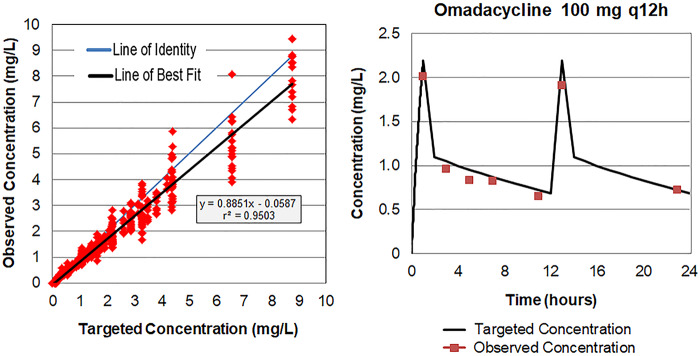
Relationship between targeted and observed omadacycline ELF concentrations simulated in the one-compartment *in vitro* infection model.

The targeted concentration-time profiles representing the beta and terminal half-lives of omadacycline in human epithelial lining fluid (ELF) were well simulated in the one-compartment *in vitro* infection model, as evidenced by the agreement between observed and targeted concentrations shown for all data evaluated in Fig. 1A and by the 100-mg every 12 h (q12h) example dosing regimen in Fig. 1B. The assessments of the mass spectrometer assay performance demonstrated that the interassay percent coefficient of variation (%CV) for the quality control samples at concentrations of 0.300, 1.05, and 8.40 μg/ml were 28.3, 9.41, and 9.09%, respectively.

Pharmacokinetic (PK) models were fit to the samples collected for the evaluation of the omadacycline concentration-time profiles. The final PK model, which was a one-compartment model utilizing two different clearance terms over the 24-h study duration, fit the data with excellent precision, as evidenced by a coefficient of determination (*r*^2^) value of 0.964 and a slope of 0.970 (which is close to 1) for the observed versus model-predicted concentrations (see Fig. S1 in the supplemental material). No bias was evident when goodness-of-fit plots were examined (data not shown). Using the final PK model, omadacycline total-drug ELF AUC values were computed through numerical integration for each dosing regimen studied in the one-compartment *in vitro* infection models, which are described in Table 3.

**TABLE 3:** Omadacycline dosing regimens and associated model-predicted total-drug ELF AUC values evaluated in the one-compartment *in vitro* infection model

Omadacycline i.v. dose studied (mg q12h)	Mean total-drug ELF AUC (mg · h/liter) (%CV)^*a*^
12.5	2.38 (0.13)
25	5.00 (13.9)
50	11.1 (14.9)
100	20.8 (12.0)
150	31.2 (10.6)
200	41.8 (10.4)
300	63.7 (12.1)
400	82.7 (12.0)

a Calculated using the model-predicted total-drug ELF concentrations from 0 to 24 h.

**FIG 2:**
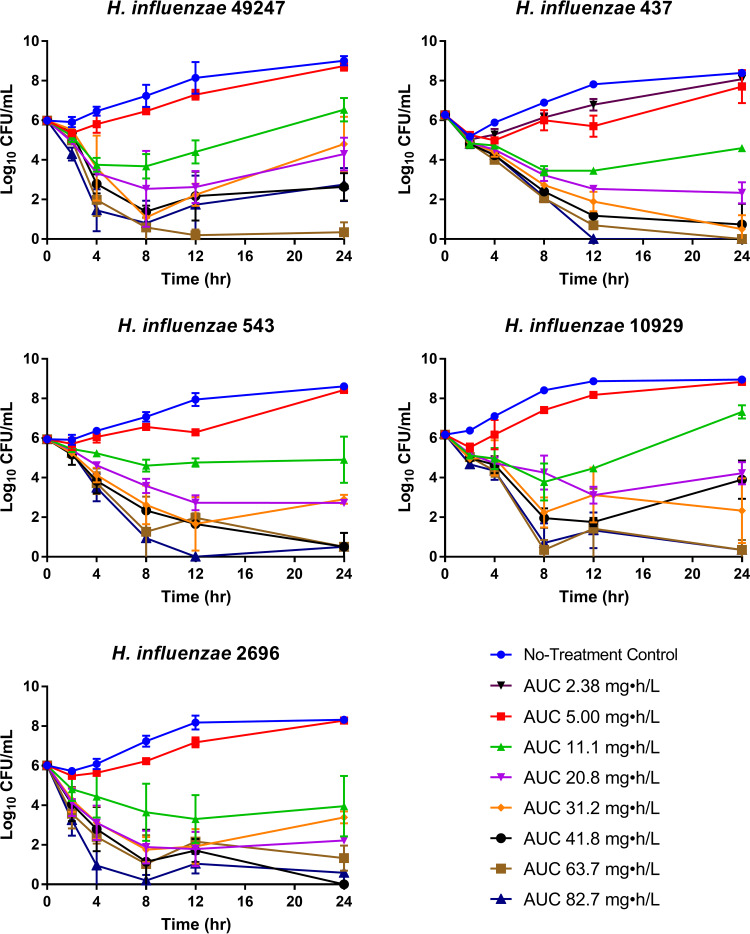
Results of dose-ranging studies carried out using the one-compartment *in vitro* infection model for the panel of five H. influenzae isolates exposed to omadacycline total-drug ELF AUC values ranging from 2.38 to 82.7 mg · h/liter, which were representative of dosing regimens of 12.5 to 400 mg q12h.

Across the omadacycline dosing regimens evaluated in the one-compartmen*t in vitro* infection model dose-ranging studies for the five H. influenzae isolates, an exposure-response relationship was observed for each isolate, as demonstrated by the time course data shown in Fig. 2. As evidenced by the data for each of the five isolates, the growth control grew well, reaching bacterial densities greater than 1.0 × 10^8^ log_10_ CFU/ml by the 24-h time point, representing a 2-log_10_ increase in bacterial burden. A range of responses was achieved over the total-drug ELF AUC range for each isolate, with low exposures resulting in treatment failure (i.e., as evidenced by the growth matching that of the control by 24 h), intermediate exposures reaching only net bacterial stasis at 24 h, and higher exposures achieving reductions in the bacterial burden over the 24-h period.

**FIG 3:**
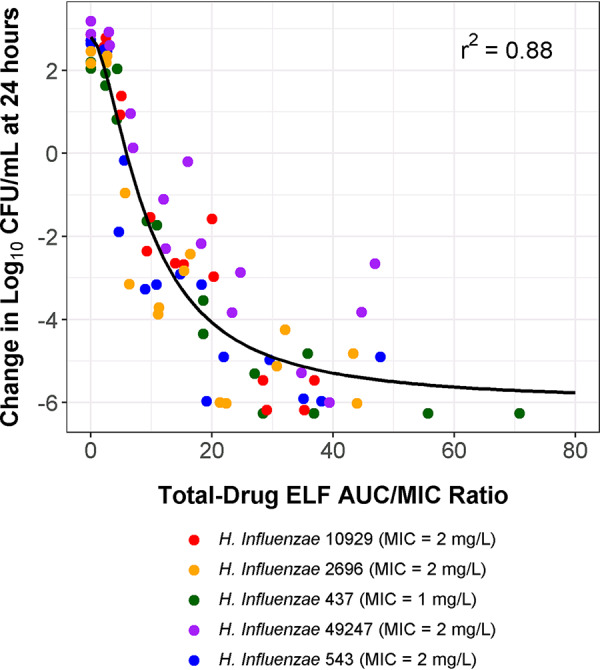
Relationship between the change in log_10_ CFU/ml from baseline at 24 h and omadacycline total-drug ELF AUC/MIC ratio based on the pooled data for the five H. influenzae isolates evaluated in the dose-ranging studies carried out using the one-compartment *in vitro* infection model.

The relationships between the change in the log_10_ CFU/ml from baseline at 24 h and omadacycline total-drug ELF AUC/MIC ratio were evaluated based on the data for each individual H. influenzae isolate as well as a pooled collection. As evidenced by a high *r*^2^ of 0.88 and the dispersion of data about the fitted function, the relationship between the change in the log_10_ CFU/ml from baseline at 24 h and total-drug ELF AUC/MIC ratio shown in Fig. 3 described the activity of omadacycline well across the panel of five H. influenzae isolates with MIC values ranging from 1 to 2 mg/liter. As shown in Table 4, the parameter estimates (standard errors) for the Hill-type model describing this relationship were as follows: the change in the log_10_ CFU/ml from baseline at 24 h in the absence of drug (*E*_0_) was 2.80 (0.34), the maximum change in the log_10_ CFU/ml from *E*_0_ (*E*_max_) was 8.81 (0.75), the Hill coefficient was 1.66 (0.29), and the omadacycline total-drug ELF AUC/MIC ratio associated with half-maximal effect (50% effective concentration [EC_50_]) was 9.29 (1.13). The magnitude of the total-drug ELF AUC/MIC ratio associated with net bacterial stasis and 1- and 2-log_10_ CFU/ml reductions from baseline at 24 h based on the pooled data were 5.87, 7.87, and 10.4, respectively.

**TABLE 4:** Summary of parameter estimates for Hill-type models and omadacycline total-drug ELF AUC/MIC ratio targets^*a*^

H. influenzae isolate	Microbroth dilution MIC (mg/liter)	Hill-type model mean parameter estimate (SE)					Total-drug ELF AUC/MIC ratio target by bacterial reduction endpoint		
		*E*_0_	*E*_max_	EC_50_	*H*	Hill-type model *r*^2^	Net bacterial stasis	1-log_10_ CFU/ml reduction from baseline	2-log_10_ CFU/ml reduction from baseline
ATCC 49247	2	3.16 (0.73)	8.20 (1.77)	11.5 (3.17)	1.74 (0.76)	0.89	8.76	11.6	15.5
437	1	2.14 (0.31)	8.60 (0.62)	11.5 (1.09)	2.16 (0.41)	0.98	6.91	8.91	11.1
543	2	2.96 (0.67)	8.55 (1.09)	6.25 (1.23)	1.86 (0.59)	0.93	4.45	5.78	7.45
2696	2	2.66 (0.75)	7.67 (1.02)	5.64 (1.06)	2.49 (1.01)	0.90	4.38	5.44	6.72
10929	1	3.07 (0.64)	12.6 (5.16)	17.7 (11.9)	1.24 (0.51)	0.94	7.09	9.73	12.9
Pooled		2.80 (0.34)	8.81 (0.75)	9.29 (1.13)	1.66 (0.29)	0.88	5.87	7.87	10.4

Mean (SD)							6.32 (1.88)	8.30 (2.64)	10.7 (3.69)
Median							6.91	8.91	11.1

a Total-drug ELF AUC/MIC ratio targets shown are those associated with various levels of bacterial reduction from baseline for H. influenzae isolates studied in the one-compartment *in vitro* infection model. *H*, Hill coefficient.

Hill-type models describing the relationship between the change in the log_10_ CFU/ml from baseline at 24 h and the total-drug ELF AUC/MIC ratio for each H. influenzae isolate also described the data well (*r*^2^ values of 0.89 to 0.94). Parameter estimates (standard errors) for these Hill-type models are shown in Table 4. The median (minimum, maximum) omadacycline total-drug ELF AUC/MIC ratio associated with net bacterial stasis and the 1- and 2-log_10_ CFU/ml reductions from baseline at 24 h were 6.91 (4.38, 8.76), 8.91 (5.44, 11.6), and 11.1 (6.72, 15.5), respectively.

## DISCUSSION

The studies described herein were carried out to accomplish two objectives. The first was to evaluate the frequency of mutation of clinical H. influenzae isolates to omadacycline. The second objective was to determine the magnitude of the omadacycline AUC/MIC ratio associated with efficacy for the above-described panel of H. influenzae isolates evaluated using a one-compartment *in vitro* infection model.

The frequency of mutation to omadacycline for the H. influenzae isolates evaluated could not be identified for bacterial populations less than or equal to 2.9 × 10^9^ CFU/ml at omadacycline concentrations representing 3 or 5 times the baseline MIC. These values are similar to those identified by Clark et al. (29) and Min et al. (30) using a variety of agents against H. influenzae. Mutation frequency values like those described herein are favorable compared to those of drug-organism combinations such as fosfomycin and Escherichia coli, the mutation frequency for which is approximately 1 × 10^5^ to 1 × 10^6^ CFU/ml (31). For drug-organism combinations with mutation frequencies in this range, the probability of on-therapy emergence of resistance is expected to be higher than those with lower mutation frequencies. While data from these 24-h studies assessing mutation frequency are useful, studies using a hollow-fiber *in vitro* infection model, a system that allows for the evaluation of the resistance suppression over a longer study period, are beneficial for characterizing the potential for development of on-therapy resistance and the identification of exposure that prevents resistance amplification.

Results obtained from the dose-ranging studies demonstrated that the data for the five isolates comodeled well. As evidenced by a high *r*^2^ of 0.88 and the dispersion of data about the fitted function, the relationship between the change in the log_10_ CFU/ml from baseline at 24 h and total-drug ELF AUC/MIC ratio described the efficacy of omadacycline against the panel of H. influenzae isolates well. Hill models describing the relationship for individual isolates also described these data well (*r*^2^ values of 0.89 to 0.94). The median omadacycline total-drug ELF AUC/MIC ratio associated with net bacterial stasis and the 1- and 2-log_10_ CFU/ml reductions from baseline at 24 h for the H. influenzae isolates studied were 6.91, 8.91, and 11.1, respectively. As described above, PK-PD targets for H. influenzae were characterized using an *in vitro* infection model, given the difficulty of achieving sufficient growth and potential for auto-clearance of the pathogen in *in vivo* infection models. Thus, while information derived from *in vitro* infection models represents an optimal approach to characterize the PK-PD targets for H. influenzae efficacy, such targets may be overestimated relative to what is expected using an *in vivo* infection model as a result of the enhanced effect of growth media on bacterial growth and the lack of an immune system.

The evaluation of effect site exposures, which for CABP is total-drug ELF, and thus the characterization of total-drug ELF AUC/MIC ratio targets represented an important strength of the studies described herein. Mimicking the shape of total-drug ELF, which involved applying two different half-live estimates observed in healthy volunteers (32), was easier to accomplish in an *in vitro* model than would have been possible using a standard murine infection model.

In the current era of drug development for antimicrobial agents, PK-PD assessments are carried out during all stages of drug development (27, 33–37). Evaluation of PK-PD targets for efficacy from nonclinical infection models, together with phase 1 PK data and Monte Carlo simulation, prior to the execution of clinical trials with patients ensures that efficacious dosing regimens are selected for studying infected patients and serves to make the drug development program less prone to risk. In late-stage development, the use of such data provides the basis for refining dosing regimens, especially in special populations, and supports decisions for interpretive criteria for *in vitro* susceptibility testing for the antimicrobial agent against a given pathogen (27). Given the empirical nature of the treatment of patients with CABP, it is important that the approved omadacycline dosing regimen for CABP be optimized from a PK-PD perspective for key pathogens, such as H. influenzae. The data described herein, which allowed for the characterization of the PK-PD of omadacycline against H. influenzae, were used to support both omadacycline dose selection for patients with CABP and evaluations for interpretive criteria for *in vitro* susceptibility testing for omadacycline against H. influenzae (38).

## MATERIALS AND METHODS

### Bacteria and study drug.

A panel of five H. influenzae isolates was utilized in the studies described herein and was obtained from the American Type Culture Collection (ATCC) and JMI Laboratories (North Liberty, IA). The challenge panel included four clinical isolates and one ATCC reference strain (H. influenzae ATCC 49247). Omadacycline was provided by Paratek Pharmaceuticals, Inc. (King of Prussia, PA).

### Media and *in vitro* susceptibility studies.

Omadacycline MIC values were determined for the challenge isolate panel according to Clinical and Laboratory Standards Institute guidelines (39) using agar and broth microdilution methodologies. These different assays were used as described below. All MIC values were determined using freshly prepared *Haemophilus* test medium (HTM), in triplicate, over a 2-day period and are reported as the modal values.

### Frequency of resistance studies.

The frequency of omadacycline resistance was estimated for each challenge isolate. In brief, 2 ml of log-phase growth suspension, consisting of approximately 1.0 × 10^9^ CFU/ml, was plated using 200-μl volumes on 10- by 100-mm HTM agar plates supplemented with three times or five times the omadacycline baseline agar dilution MIC. The bacterial concentration in the suspension was determined by quantitative culture using drug-free agar. The inoculated agar plates were incubated at 35°C and then observed for growth, with individual colonies counted 48 h postincubation. The frequency of resistance was determined as the ratio of the number of colonies found growing on the drug-supplemented agar plates to the total number of colonies plated. If no isolates were observed after 48 h of incubation, then the resultant mutation frequency was determined to be less than that of the inoculum evaluated.

### One-compartment *in vitro* infection model.

A one-compartment *in vitro* infection model, as previously described (40, 41), was used for the studies described herein. In brief, the *in vitro* model consisted of a central infection compartment, which contained growth medium (HTM), the challenge organism, and magnetized stir bars to ensure that the test compound, media, and organism remained in a homogenous state. The central infection compartment was placed on a magnetic stir plate, which was then housed in a temperature-controlled incubator set to 35°C. The bacterial suspension in the central compartment was then exposed to dynamic concentration-time profiles of omadacycline designed to simulate human ELF concentration-time profiles in healthy volunteers following i.v. administration (32). A series of computer-controlled peristaltic pumps infused drug-free media into the central compartment, while simultaneously removing media into a waste container. The challenge isolate was then inoculated directly into the central infection compartment, and the test compound was infused by a computer-controlled syringe pump in order to simulate selected beta- and terminal half-lives, dosing frequencies, and durations of infusion, as described below. The diffusion rates simulated by the peristaltic pumps were such that the desired concentration-time profile of omadacycline imitated human total-drug ELF concentration-time profiles. Samples were aseptically collected from the central compartment for determination of bacterial density and drug concentration at the predetermined time points described below.

The bacterial suspension of each challenge isolate was prepared from a culture grown overnight on chocolate II agar plates, purchased from BD Laboratories (Franklin Lakes, NJ). Isolates were collected from the overnight cultures, suspended in HTM broth, and grown to mid-logarithmic growth phase in an Erlenmeyer flask immersed in a shaking water bath set to 35°C and 125 rotations per minute. The optical density of the bacterial suspension growing in the flask was compared to those of previously completed growth curves using a spectrophotometer set to a wavelength of 630 nm. Isolates were then inoculated in the system to achieve an initial bacterial burden of 1.0 × 10^6^ CFU/ml. The bacterial suspension in the central compartment was then exposed to changing omadacycline concentrations, simulating human beta and terminal half-lives of 1 and 14.9 h in total-drug ELF (32). Omadacycline dosing regimens of 12.5, 25, 50, 100, 150, 200, 300, and 400 mg administered every 12 h were linearly scaled based on ELF concentrations observed following a 100-mg i.v. dose. All omadacycline treatment regimens were compared to a no-treatment control.

In order to evaluate the effect that omadacycline had on the bacterial suspension in the central compartment, a series of 1-ml samples were collected for the determination of bacterial density at 0, 2, 4, 8, 12, and 24 h. In order to eliminate the potential for drug carryover, each bacterial sample was centrifuged, aspirated, and resuspended to the initial sample volume with sterile normal saline in duplicate to eliminate drug carryover. The washed sample was diluted serially by 10-fold volumes in sterile normal saline, cultured on chocolate agar, and placed in a humidified incubator for 24 h. In order to measure drug concentrations in the *in vitro* system, a second 1-ml sample aliquot was collected at 1, 2, 3, 5, 7, 11, 13, and 23 h postinoculation. All samples for evaluation of omadacycline concentration were immediately frozen at –80°C until they were assayed via liquid chromatography-tandem mass spectrometry (LC/MS/MS).

### Drug assay.

Calibration standard and quality control samples were prepared in HTM and processed concurrently with collected samples. All samples were assayed via LC/MS/MS on a Sciex 5500 mass spectrometer using a Thermo Hypercil Gold C8 column (3 by 100 mm) with a mobile phase of 10 mM ammonium formate (pH 3.0) and acetonitrile. The standard curve for omadacycline was linear, ranging from 0.10 to 10.0 mg/liter, with a correlation coefficient (*r*^2^) value ranging from 0.9926 to 0.9992. The lower limit of quantification was 0.100 mg/liter.

### PK-PD analyses.

PK models were fit to the samples collected for the evaluation of the total-drug ELF concentration-time profiles. Omadacycline total-drug ELF AUC values were calculated for each dosing regimen simulated in the one-compartment *in vitro* infection model. Given the assumption that protein binding in ELF is negligible, total-drug ELF AUC values were evaluated and not further adjusted to account for protein binding. Data from the omadacycline dose-ranging studies were evaluated using Hill-type models and nonlinear least-squares regression. All data were weighted using the inverse of the estimated measurement variance. The relationship between the change in log_10_ CFU/ml from baseline at 24 h and total-drug ELF AUC/MIC ratio was evaluated by utilizing the broth dilution MIC values. The magnitude of the total-drug AUC/MIC ratio associated with net bacterial stasis and 1- and 2-log_10_ CFU/ml reductions from baseline at 24 h was determined for each H. influenzae isolate individually and based on the pooled data for the isolates studied.

## Supplementary Material

Supplemental file 1
