# Supplementary material for: Pharmacokinetic-Pharmacodynamic Characterization of Omadacycline against Haemophilus influenzae Using a One-Compartment In Vitro Infection Model
Source: Antimicrob Agents Chemother. 2020 May 21;64(6):e02265-19. doi: 10.1128/AAC.02265-19 (PMC7269464; doi:10.1128/AAC.02265-19)

Figure S1. Relationship between observed and model-predicted total-drug ELF omadacycline concentrations

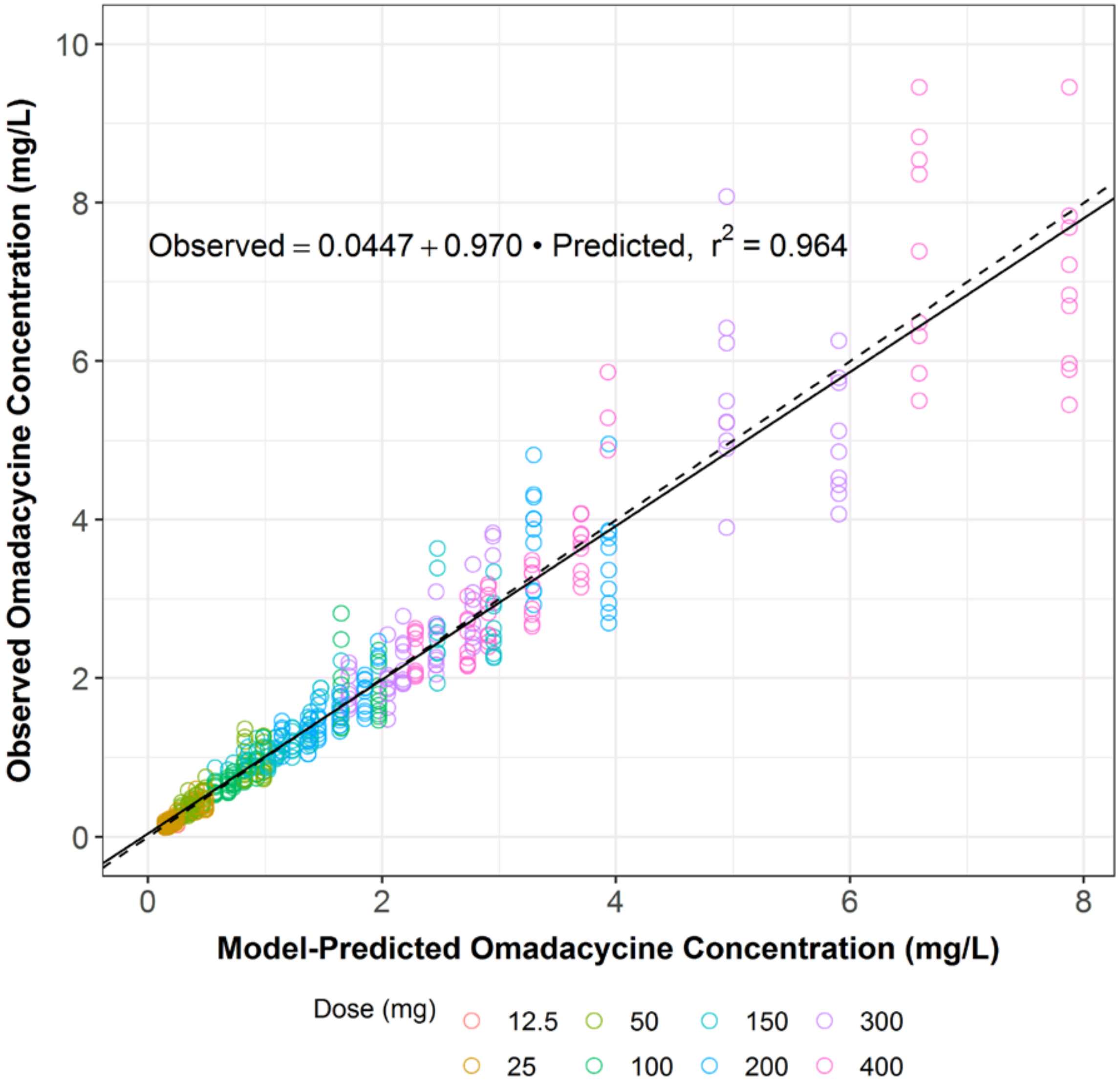

Supplement: Supplemental file 1 [file AAC.02265-19-s0001.pdf]
